# Clinical, Molecular, and Functional Characterization of *CLCN1* Mutations in Three Families with Recessive Myotonia Congenita

**DOI:** 10.1007/s12017-015-8356-8

**Published:** 2015-05-26

**Authors:** Simona Portaro, Concetta Altamura, Norma Licata, Giulia M. Camerino, Paola Imbrici, Olimpia Musumeci, Carmelo Rodolico, Diana Conte Camerino, Antonio Toscano, Jean-François Desaphy

**Affiliations:** Section of Pharmacology, Department of Pharmacy and Drug Sciences, University of Bari Aldo Moro, Via Orabona 4 – Campus, 70125 Bari, Italy; Department of Neurosciences, University of Messina, Messina, Italy

**Keywords:** Myotonia congenita, Molecular analysis, *CLCN1* gene, Functional characterization, TSA cells, RT-PCR analysis

## Abstract

Myotonia congenita (MC) is an inherited muscle disease characterized by impaired muscle relaxation after contraction, resulting in muscle stiffness. Both recessive (Becker’s disease) or dominant (Thomsen’s disease) MC are caused by mutations in the *CLCN1* gene encoding the voltage-dependent chloride ClC-1 channel, which is quite exclusively expressed in skeletal muscle. More than 200 *CLCN1* mutations have been associated with MC. We provide herein a detailed clinical, molecular, and functional evaluation of four patients with recessive MC belonging to three different families. Four *CLCN1* variants were identified, three of which have never been characterized. The c.244A>G (p.T82A) and c.1357C>T (p.R453W) variants were each associated in compound heterozygosity with c.568GG>TC (p.G190S), for which pathogenicity is already known. The new c.809G>T (p.G270V) variant was found in the homozygous state. Patch-clamp studies of ClC-1 mutants expressed in tsA201 cells confirmed the pathogenicity of p.G270V, which greatly shifts the voltage dependence of channel activation toward positive potentials. Conversely, the mechanisms by which p.T82A and p.R453W cause the disease remained elusive, as the mutated channels behave similarly to WT. The results also suggest that p.G190S does not exert dominant-negative effects on other mutated ClC-1 subunits. Moreover, we performed a RT-PCR quantification of selected ion channels transcripts in muscle biopsies of two patients. The results suggest gene expression alteration of sodium and potassium channel subunits in myotonic muscles; if confirmed, such analysis may pave the way toward a better understanding of disease phenotype and a possible identification of new therapeutic options.

**Electronic supplementary material:**

The online version of this article (doi:10.1007/s12017-015-8356-8) contains supplementary material, which is available to authorized users.

## Introduction

Clinical myotonia impairs muscle relaxation after voluntary intense contraction. Myotonia congenita (MC) is an inherited myotonia due to mutations in the *CLCN1* gene encoding the skeletal muscle ClC-1 chloride channel (Koch et al. 1992; George et al. 1993). Loss-of-function mutations of ClC-1 channel reduce the sarcolemmal chloride conductance, which, in turn, increases sarcolemma excitability and causes a delayed relaxation manifesting as a clinical and electrical myotonia (Imbrici et al. 2015). From the clinical view, MC patients usually describe muscle stiffness after initiating a forceful movement (Lossin and George 2008). Both dominant and recessive inheritance patterns are found in MC families. Becker myotonia congenita, the recessive form, is typically more severe and has an earlier onset than the dominant one, Thomsen myotonia congenita (TMC). TMC often has a wider range of presentations, including subclinical to moderately severe forms. Consequently, these two entities may be distinguished by inheritance pattern, age at onset, and phenotype (Lossin and George 2008; Heatwole et al. 2013).

To date, more than 200 pathogenic mutations have been reported in the *CLCN1* gene, being widely distributed across the 23 exons or within introns (Pusch et al. 1995; Lossin and George 2008; Mazón et al. 2012; Raja Rayan et al. 2012; Brugnoni et al. 2013; the Human Gene Mutation Database). A number of mutations resulting in premature stop codons are not expected to yield functional proteins, but these may variously affect phenotypes (Richardson et al. 2014). Splicing mutations have also been reported, causing out-of-frame mRNA transcripts that do not produce functional ClC-1 (Ulzi et al. 2014). A number of missense mutations have been functionally characterized by measuring chloride currents generated by mutant channels heterologously expressed in cell lines. These studies have been critical to better understand the relationship between ClC-1 channel structure and function.

Experimental studies have demonstrated that *CLCN1* gene mutations can lead to a positive shift of the activation curve, a reduced chloride ion permeation, an increased cation permeability, an inverted voltage dependence, or a defect in protein trafficking (Imbrici et al. 2015). All these alterations reduce the activity of ClC-1 channel mutants, leading to a reduced sarcolemmal chloride conductance. Functional studies have also revealed possible differences between recessive and dominant mutations. The ClC-1 channel is a homodimer with each subunit forming a single pore; the two parallel pores can gate independently (fast gates), while a common slow gate can close both pores together (Saviane et al. 1999). This peculiar structure may explain the two MC inheritance traits (Pusch et al. 1995). A recessive mutation is expected to induce loss of function of the sole mutated subunit. The coexpression of the recessive mutation with the wild-type ClC-1 results at maximum in a 50 % reduction of the sarcolemmal chloride conductance, which is not enough to cause myotonia. The presence of the recessive mutation in homozygosity or two mutations in compound heterozygosity is required to reduce the sarcolemmal chloride conductance by >50 % and to induce myotonia. In contrast, a dominant mutation is expected to exert an adverse effect on the associated wild-type subunit (the so-called dominant-negative effect), which is sufficient to reduce the sarcolemmal chloride conductance by >50 %, so inducing myotonia. Although dominant mutations may show full penetrance, a dominant inheritance pattern with incomplete penetrance was observed in some pedigrees (Plassart-Schiess et al. 1998). The situation may be even more complicated, since some mutations may be recessive in some pedigrees or dominant in others, suggesting that background modifying factors may greatly contribute to the variability of myotonia.

We report, herein, the clinical, molecular, and functional study of four individuals belonging to three different families affected by recessive MC. In addition, we performed a RT-PCR quantification of selected ion channel subunits expression in muscle biopsies of two MC patients.

## Materials and Methods

### Clinical Evaluation

The study was approved by local ethics committees and conducted in accordance with the Declaration of Helsinki. We have evaluated four patients presenting with MC and belonging to three families. The patients were 20–44 years old (three males and one female). All patients were referred to our clinic because of muscle stiffness and rigidity. The Medical Research Council (MRC) scale for muscle strength was applied. Neurological examination was also specifically addressed to search for myotonic signs as tongue, eyelid, lid-lag, jaw, handgrip, and percussion myotonia. EMG study was performed according to Fournier’s guidelines (Fournier et al. 2004). Three out of the four patients (two were siblings) underwent a *Vastus Lateralis* muscle biopsy.

### Genetic Testing

After having obtained written informed consent from patients, blood samples were collected and genomic DNA was extracted according to standard methods. Molecular analysis of the *CLCN1* gene was performed by amplification and direct sequencing of all the 23 exons. PCR was carried out with 12.5 µl AmpliTaq Gold 360 MasterMix (Applied Biosystems, Forster City, CA), 10 pmol of each M13-tagged primer, and 100 ng of genomic DNA, under the following thermal cycler conditions: an initial denaturing step at 95 °C for 10 min followed by 40 cycles at 95 °C for 30 s, 58 °C for 30 s, 72 °C for 30 s and a final extension step at 72 °C for 7 min. The PCR products were purified and sequenced on AB 3500 (Applied Biosystem) (Raja Rayan et al. 2012).

### Functional Characterization

The hClC-1 mutants were obtained in pRcCMV-hClC-1 plasmid using the QuickChange™ site-directed mutagenesis kit and transiently expressed in tsA201 cells using the calcium phosphate precipitation method, as previously described (Desaphy et al. 2013). Cells were used between 36 and 80 h after transfection for whole-cell patch-clamp recording of chloride currents using Axopatch 200B amplifier and Digitata 1440A AD-DA converter (Axon Instruments). Pipettes were pulled form borosilicate glass capillaries and had ~3 MΩ resistance. Cells were bathed in extracellular solution containing (in mM) 140 NaCl, 4 KCl, 2 CaCl_2_, 1 MgCl_2_, 5 HEPES (pH = 7.4). A high-chloride pipette solution was used to record huge chloride currents and analyze current deactivation kinetics; the composition was (in mM) 130 CsCl, 2 MgCl_2_, 5 EGTA, 10 HEPES (pH 7.2). With this solution, the equilibrium potential for chloride ions was about −3 mV. Thus, the cells were clamped at 0 mV and chloride currents were elicited by 400-ms-long voltage steps ranging from −200 to +120 mV for WT, p.T82A, and p.R453W channels. Because p.G270V mutant generated low-amplitude currents, the protocol was modified to test voltage steps ranging from −120 to +200 mV. Each of the voltage steps was followed by a 400-ms-long voltage step at −105 mV, before returning to holding potential. To examine the voltage dependence of channel activation (i.e., apparent open probability), the normalized instantaneous current measured at −105 mV was plotted as a function of the voltage of preceding steps and the relationships were fitted with a Boltzmann equation [*P*_0_(*V*) = Min + (1 − Min)/(1 + exp((*V* − *V*_0.5_)/*S*))], where Min is the minimal value of *P*_0_, *V*_0.5_ is the half-maximal activation potential, and *S* is the slope factor. A low-chloride pipette solution was used to record chloride currents in a more physiological condition; it was obtained by substituting cesium chloride by cesium glutamate, and the equilibrium potential for chloride ions was close to −92 mV. In this condition, the holding potential was set at −95 mV and 400-ms-long voltage steps were applied from −150 and +150 mV. Currents were low-pass filtered at 2 kHz and digitized at sampling rates of 50 kHz. Chloride currents were recorded 5 min after achieving the whole-cell configuration to allow the complete cell filling with pipette solution. Data were analyzed off-line using pClamp 10.3 (Axon Instrument) and SigmaPlot 8.02 (Systat Software GmbH). Cells exhibiting voltage errors >5 mV after series resistance compensation or non-negligible leak currents were discarded from analysis.

### Quantitative Real-time PCR Analysis

Human *Vastus Lateralis* muscle biopsies were snap-frozen in liquid nitrogen soon after removal and stored at −80 °C until use. For each muscle sample, the total RNA was isolated by an RNeasy Fibrous Tissue Mini Kit (Qiagen C.N. 74004, Valencia) and quantified using a spectrophotometer (ND-1000 NanoDrop, Thermo Scientific, USA).

Due to the low amount of RNA obtained from the human biopsies, amplification was necessary and was performed using Ovation PicoSL WTA system V2 (NuGEN C.N. 3312, USA), as previously described (Sandonà et al. 2012). For each sample, 40 ng of total RNA was incubated at 4 °C for 5 min and 65 °C for 2 min with 2 μl of A1 solution (1° Strand primer mix, C.N. S01493, NuGEN, USA). Each sample mix was then supplemented with 2.5 μl of A2 solution (1° Strand buffer mix, C.N. S01494, NuGEN, USA) and 0.5 μl of A3 solution (1° Strand enzyme mix, C.N. S01495, NuGEN, USA) and incubated at 25 °C for 30 min, 42 °C for 15 min, and 70 °C for 15 min. To each sample mix, 9.7 μl B1 solution (2° Strand buffer mix, C.N. S01496, NuGEN, USA) and 0.3 μl B2 solution (2° Strand enzyme mix, C.N. S01377, NuGEN, USA) were added and incubated at 25 °C for 10 min, 50 °C for 30 min, and 80 °C for 20 min. Then 32 μl of beads (Agen court RNA clean XP, bealds C.CN S01307, NuGEN, USA) was added to each sample mix and incubated for 10 min at 25–30 °C. The tubes were transferred to the SPRIPlate Ring Super Magnet Plate (C.N. A32782 Beckman Coulter Genomic, USA) and incubated for 5 min. After removing 45 μl of solution, the beads were washed three times with ethanol 70 %, resuspended with 20 μl of C2 solution (SPIA buffer mix, C.N. S01498, NuGEN, USA), 10 μl of C1 solution (SPIA Primer mix, C.N. S01497, NuGEN, USA), and 10 μl of C3 solution (SPIA enzyme mix, C.N. S01499, NuGEN, USA), and incubated for 47 °C for 75 min and 95 °C 5 min. The solutions without beads were purified with QiAquick PCR purification kit (Qiagen C.N. 28104, Valencia).

All the RT-PCR experiments were performed in agreement with the MIQE guidelines for qPCR, as published (Bustin et al. 2009). Real-time PCR was performed using the Applied Biosystems Real-time PCR 7500 Fast system (USA), MicroAmp Fast Optical 96-Well Reaction Plate 0.1 μl (Life Technologies C.N. 4346906), and MicroAmp Optical Adhesive Film (Life Technologies C.N. 4311971). Each reaction was performed in triplicate on a single-plex reaction. The setup of reactions consisted in 1.2 ng cDNA, 0.5 μl of TaqMan Gene Expression Assays (Life Technologies), 5 μl of TaqMan Universal PCR master mix No AmpErase UNG (2x) (Life Technologies C.N. 4324018), and nuclease-free water (not diethylpyrocarbonate (DEPC) treated) (Life Technologies C.N. AM9930) for a final volume of 10 μl. The RT-TaqMan PCR conditions were as follows: step 1: 95 °C for 20 s, step 2: 95 °C for 3 s, and step 3: 60 °C for 30 s; steps 2 and 3 were repeated 40 times. The results were compared with a relative standard curve obtained from six points of 1:4 serial dilutions. The mRNA expression of the genes was normalized to the housekeeping gene β-actin, which was more stable as compared to β2-microglobulin (β2m) and hypoxanthine phosphoribosyltransferase 1 (Hprt1). TaqMan hydrolysis primer and probe gene expression assays were ordered from Life Technologies with the assay IDs reported in Online Resource 1.

Because the potassium voltage-gated channel, Isk-related family, member 3, also known as MinK-related protein type 2 (MiRP2), and encoded by the *KCNE3* gene, showed very low level of expression, a pre-amplification by TaqMan PreAmp Master Mix (Life Technologies C.N. 4391128) was performed before the RT-PCR experiment. The setup of pre-amplification consisted in 250 ng of cDNA (in 12.5 ml volume), 25 μl of TaqMan PreAmp Master Mix (2×), and 12.5 μl of pool assay 0.2× (containing Kcne3, Hprt1, β2m and β-actin). The solution was incubated at 50 °C for 2 min, 95 °C for 10 min, 95 °C for 15 s for 40 cycles, and 60 °C for 1 min.

## Results

### Case Reports

**Fig. 1:**
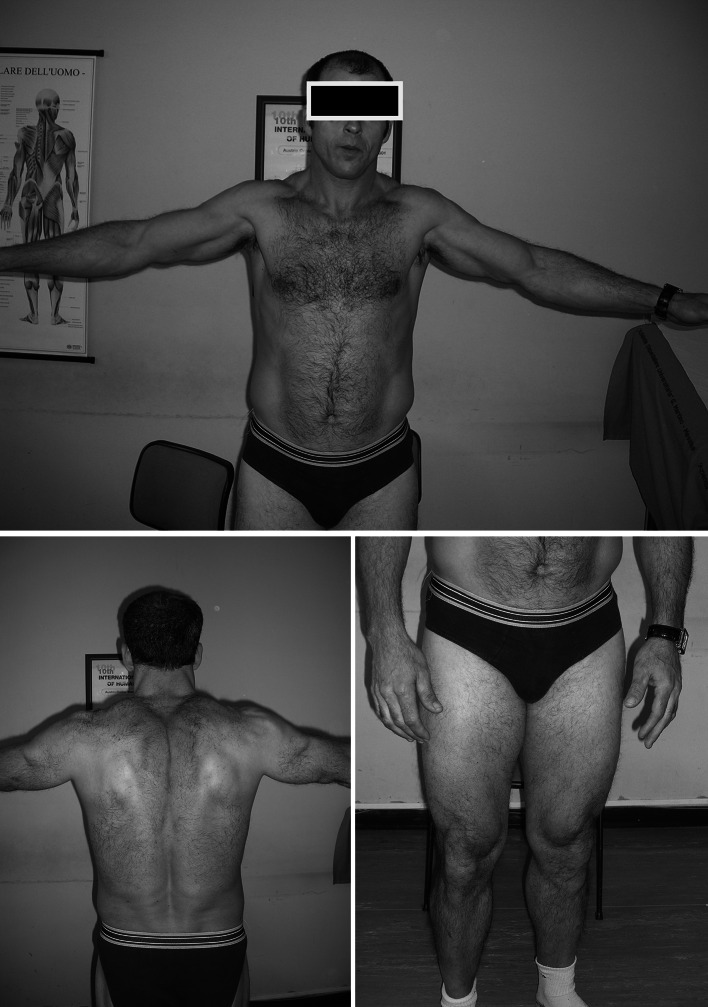
Pictures of family 1 brother showing generalized muscle hypertrophy at shoulder girdle and lower limbs

**Fig. 2:**
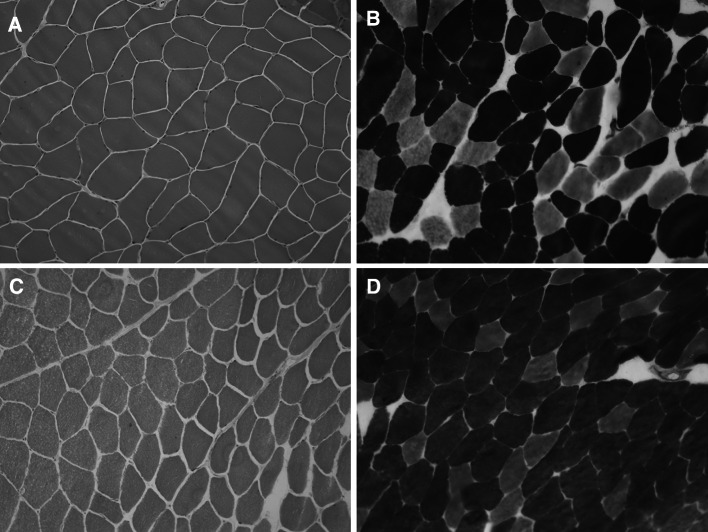
Hematoxylin and eosin (H&E) stain and ATPase stain of *Vastus Laterali*s muscle biopsies from family 1 brother (**a**, **b**) and family 3 proband (**c**, **d**) suggest muscle fiber size variability and type 2 fibers predominance

Family 1 presented with two siblings, a male of 44 years and a female of 39 years, born from consanguineous parents. Family history was negative for neuromuscular disorders. Pregnancy and birth circumstances were unremarkable as well as psychomotor development. Since the age of 10 years, both referred easy fatigability, muscle stiffness at lower limbs, and marked difficulty in climbing stairs. The sister also complained of intense muscle aches at the lower limbs and cramps. She was affected by hypothyroidism, high blood pressure, and tachyarrhythmia. The CK levels were slightly increased (slightly lower than 400 U/l − normal value: <200 U/l). Her brother showed an early baldness and complained of diffuse myalgia and muscle stiffness with initially delayed motor activities, mainly after a period of rest. These symptoms were exacerbated by cold temperature. His CK levels were elevated (up to 1200 U/l). Over the time, each sibling developed a progressive muscle stiffness involving also the upper limbs, with difficulty in opening fist or eyelids, chewing, and starting to speak. Both presented with a generalized muscle hypertrophy, more evident in the male (Fig. 1). A “warm-up phenomenon” in eyelid, handgrip, jaw, and percussion myotonia was evident in both siblings. EMG studies disclosed myotonic discharges in all examined muscles. A muscle biopsy, performed in the male, showed scattered areas of hypotrophic and hypertrophic fibers with no increase in endomysial connective tissue and type 2 fibers predominance and hypotrophy (Fig. 2a, b). Molecular genetic testing revealed a compound heterozygosity in both siblings with c.568GG>TC (p.G190S) and c.244A>G (p.T82A) changes in the *CLCN1* gene.

In family 2, the proband was a male first examined at the age of 24 years. No family history for neuromuscular disorders was reported. The patient referred the onset of symptoms at 21 years of age, characterized by tendency to fall down because of sudden muscle stiffness and weakness at lower limbs. Since then, he complained of easy fatigue, myalgia, muscle cramps, mainly at trunk and lower limbs. He also referred difficulties in opening the fist, starting movements, or climbing stairs. All these symptoms were worsened by cold temperature. Clinical examination revealed generalized muscle hypertrophy as well as eyelid, jaw, tongue, and handgrip myotonia. He complained of bilateral keratoconus. EMG showed myotonic discharges with multiple runs of spontaneous positive wave discharges that varied in both frequency and amplitude and lasted approximately 2 to 3 s. Muscle biopsy showed unspecific features as muscle fiber size variability and increased central nuclei. DNA sequencing showed a compound heterozygous condition with c.1357C>T (p.R453W) and c.568GG>TC (p.G190S) changes in the *CLCN1* gene.

The proband of family 3 was born from non-consanguineous parents. His family history was positive for neuromuscular disorders: His mother and maternal grandfather presented a generalized muscle stiffness and difficulties in opening hands (they refused to perform any further clinical and laboratory examinations). Since birth, the patient presented delayed muscle relaxation, but no painful contractures induced by cold or by emotional stress were recorded. Since the age of 25 years, he complained of muscle rigidity and difficulties in starting chewing, speaking, and walking. He showed generalized muscle hypertrophy as well as eyelid, handgrip, jaw, lid-lag and percussion myotonia with a warm-up phenomenon. CK levels were normal; EMG showed myotonic discharges in all examined muscles. His muscle biopsy disclosed slight fiber size variability and type 2 fibers predominance (Fig. 2c, d). The DNA sequencing showed homozygosity for the c.809G>T (p.G270V) change in the *CLCN1* gene.

### Functional Characterization of p.T82A, p.G270V and p.R453W Variants

Using clustal2.2, multiple amino acid sequence alignment indicated that p.T82 is conserved in rat ClC-1 and substituted for by p.M82 in mouse and dog ClC-1 (Online Resource 2). However, it is poorly conserved among human CLC protein isoforms. The p.G270 and p.R453 residues are well conserved among ClC-1 of mammals and various human CLC proteins. As previously reported, the p.G190 residue is well conserved among CLC proteins (Ulzi et al. 2012; Desaphy et al. 2013). Positioning of the mutations in the 3D structural model of the CLC-ec1 protein of *Escherichia coli* suggests that p.T82A is located at the cytoplasmic face of the channel, while p.R453W is on the external side (Online Resource 3). Both p.G190S and p.G270V are located deep inside the protein, in close proximity to the chloride conduction pathway (Dutzler 2006).

**Fig. 3:**
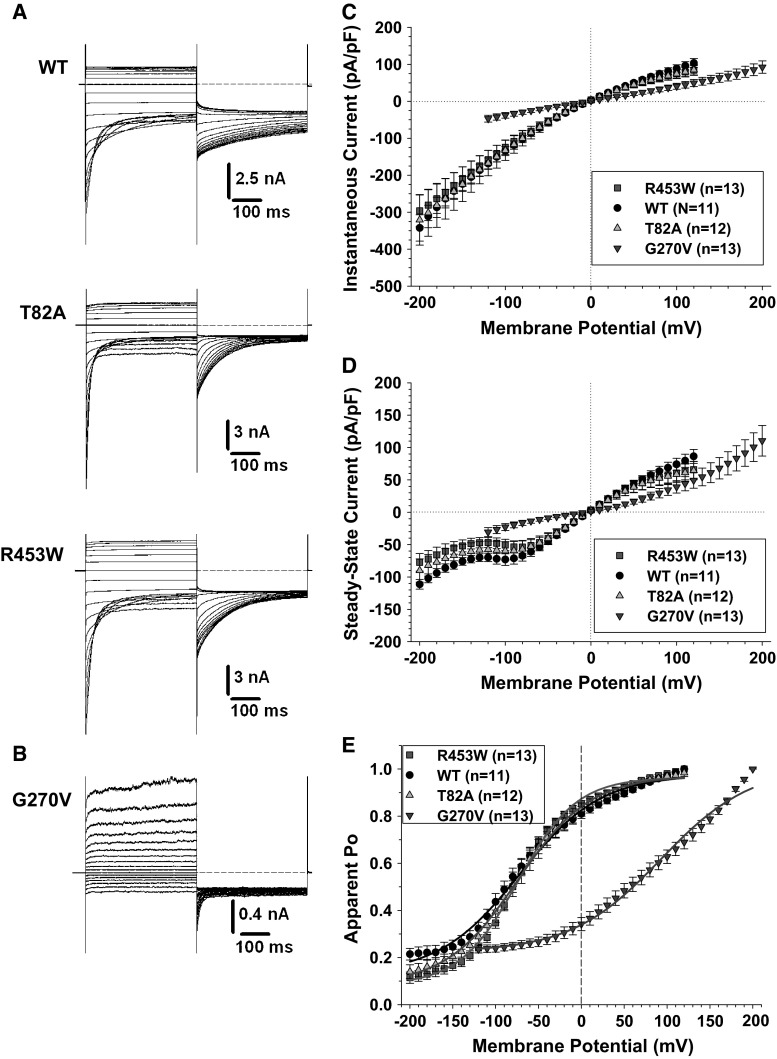
Chloride currents generated by wild-type hClC-1 channels and MC ClC-1 variants in high intracellular chloride condition. **a** Typical chloride currents recorded in HEK293 cells transfected with wild-type, p.T82A, or p.R453W hClC-1 variants. Cells are held at 0 mV, and 400-ms voltage pulses are applied from −200 to +120 mV in 10-mV intervals every 3 s. For clarity, only current traces obtained every 20 mV are shown. **b** Voltage pulses are applied from −120 to +200 mV to elicit chloride currents in HEK293 cells expressing p.G270V hClC-1 variant. **c** The instantaneous currents are measured at the beginning of test voltage pulses, normalized with respect to cell capacitance (pA/pF), and reported as a function of voltage. Each point is the mean ± SEM from 11 to 13 cells. Similar current density and strong inward rectification are observed for WT, p.T82A, and p.R453W channels. The relationship for p.G270V channels is linear. **d** Steady state currents are measured at the end of test voltage pulses and reported as mean current density ±SEM in function of voltage. Again, relationships for WT, p.T82A, and p.R453W channels are very similar, whereas current density and rectification are different for p.G270V. **e** The voltage dependence of activation is determined by plotting the apparent open probability (Po), calculated from tail currents measured at −105 mV, as a function of test voltage pulses. The relationships obtained from averaged data are fitted with a Boltzmann equation, and fit parameters are reported in Table 1. The activation curves for WT, p.T82A, and p.R453W are superimposed, whereas p.G270V channels displayed voltage dependence greatly shifted toward positive voltages

Recent studies have functionally characterized the p.G190S hClC-1 channel mutant (Ulzi et al. 2012; Desaphy et al. 2013). Here, we studied the chloride currents generated by p.T82A, p.G270V, and p.R453W hClC-1 channel mutants in transfected tsA201 cells (Fig. 3). The p.T82A and p.R453W currents were very similar to WT current, showing instantaneous currents at each voltage steps, which decreased over time between −200 and −60 mV (corresponding to current deactivation) or remained stable within 400 ms between −60 and +120 mV (Fig. 3a). Current amplitude saturated at voltages greater than +50 mV. In contrast, the p.G270V currents slowly activated at voltages more greater than +50 mV, and no amplitude saturation was observed up to +200 mV (Fig. 3b). Accordingly, while the I–V relationships for instantaneous and steady state current densities were merely superimposed for WT, p.T82A, and p.R453W, current densities of p.G270V mutant were greatly reduced along the physiological voltage range (Fig. 3c, d). The voltage dependence of channel activation (i.e., apparent open probability) was examined by plotting the normalized instantaneous current measured at −105 mV as a function of the voltage of preceding steps (Fig. 3e). Because p.G270V currents did not saturate, the maximal value for normalization was arbitrarily taken at +200 mV. The Boltzmann parameters are reported in Table 1. No difference was found between WT, p.T82A, and p.R453W channels. In contrast, the voltage dependence of p.G270V was greatly shifted toward positive values.

**Table 1:** Boltzmann parameters of activation relationships of wild-type hClC-1 channel and MC variants

[Cl−]_*i*_	Variants	*N*	*V*_0.5_ (mV)	*S* (mV)	Min
134 mM	WT	11	−72.0 ± 3.3	47.2 ± 3.3	0.13 ± 0.02
	p.T82A	12	−74.2 ± 1.6	42.2 ± 1.5	0.08 ± 0.01
	p.R453W	13	−73.8 ± 1.7	34.1 ± 1.6	0.08 ± 0.01
	p.G190S	9	+116.2 ± 3.1	33.1 ± 2.1	0.11 ± 0.01
	p.G270V	13	+92.7 ± 5.9	52.6 ± 5.1	0.22 ± 0.01
	p.T82A + p.G190S	9	−22.1 ± 2.5	69.3 ± 2.9	0.06 ± 0.02
	p.R453W + p.G190S	6	+45.2 ± 1.9	56.4 ± 1.8	0.14 ± 0.01
4 mM	WT	14	−14.8 ± 2.4	43.5 ± 2.7	<0.01
	p.T82A	17	−4.1 ± 2.1	40.3 ± 2.3	<0.01
	p.R453W	12	−8.6 ± 3.0	56.3 ± 3.8	0.03 ± 0.01
	p.G270V	13	+84.4 ± 14.2	45.8 ± 8.3	0.07 ± 0.02

The averaged activation relationships obtained from n cells are fitted with a Boltzmann equation [*P*_0_(*V*) = Min + (1 − Min)/(1 + exp((*V* − *V*_0.5_)/*S*)], where *V*_0.5_ is the half-maximal activation potential, *S* is the slope factor, and Min is the minimal value of *P*_0_. The parameters are expressed as the calculated fit value ± the standard error of the fit

We analyzed the kinetics of current deactivation for WT, p.T82A, and p.R453W channels as a function of voltage in high-chloride condition. Deactivating currents were fitted with a double exponential function including a residual current. No difference was found between the three channels in the two time constants and in the relative amplitude of the fast, slow, and non-deactivating components (Online Resource 4).

Chloride currents were also measured using a low-chloride pipette solution, to mimic the physiological equilibrium potential for chloride ions (Online Resource 5). Slowly activating outward currents were recorded at voltages greater than −50 mV for WT, p.T82A, and p.R453W channels and greater to 0 mV for p.G270V. Current density at steady state was similar within the entire voltage range for WT, p.T82A, and p.R453W channels, whereas it was greatly reduced for p.G270V between −50 and +100 mV. The voltage dependence of channel activation was fitted with a Boltzmann equation (Table 1). The relationships were superimposed for WT, p.T82A, and p.R453W channels, whereas the voltage dependence of p.G270V was greatly shifted toward positive voltages.

**Fig. 4:**
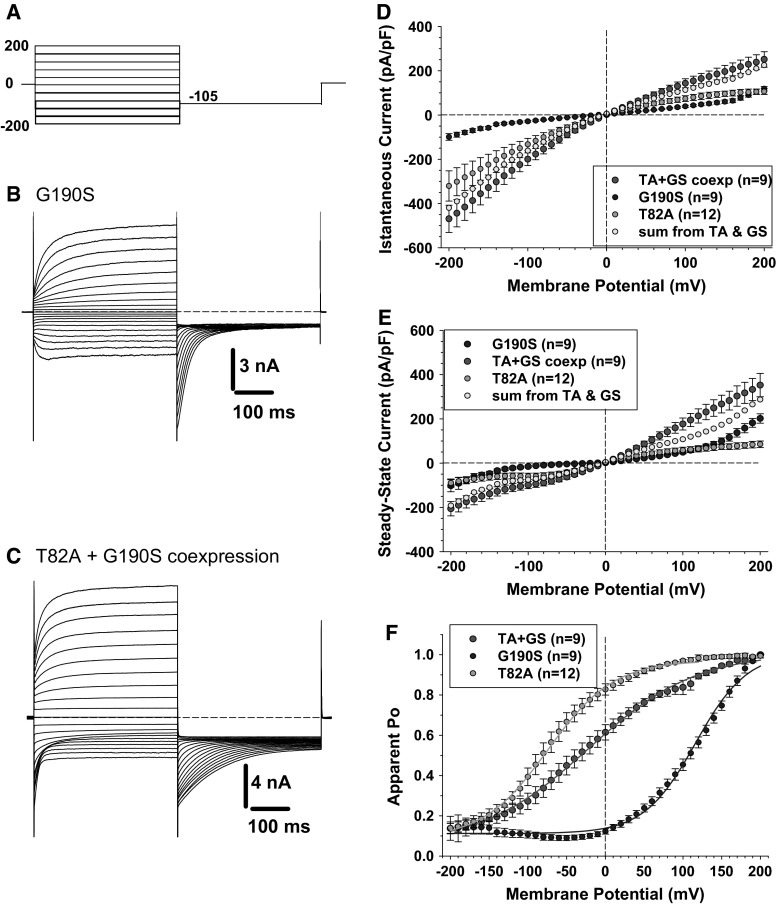
Chloride currents generated by p.G190S and coexpressed p.G190S and p.T82A mutants. **a** Chloride currents are recorded in HEK293 cells using high intracellular chloride condition. The cells are held at 0 mV, and voltage pulses are applied from −200 to +200 mV in 10-mV intervals every 3 s. For clarity, only current traces obtained every 20 mV are shown. **b** Typical chloride currents recorded in HEK293 cells transfected with 5 µg of p.G190S mutant. **c** Typical chloride currents recorded in HEK293 cells cotransfected with 5 µg of p.G190S and 5 µg of p.T82A. **d** The instantaneous currents are measured at the beginning of test voltage pulses, normalized with respect to cell capacitance (pA/pF), and reported as a function of voltage. Each point is the mean ± SEM from 9 to 12 cells. The mean I–V curves are shown for p.T82A, p.G190S, and coexpressed p.G190S + p.T82A. In *yellow* are reported the algebraic sum of current densities calculated for p.G190S and p.T82A expressed alone. The *yellow points* are quite superimposed to the I–V relationship for coexpressed p.G190S + p.T82A. **e** Steady state currents are measured at the end of test voltage pulses and reported as mean current density ±SEM in function of voltage. Again, the *yellow points* are quite superimposed to the I–V relationship for p.G190S + p.T82A coexpression. **f** The voltage dependence of activation is determined by plotting the apparent open probability (Po), calculated from tail currents measured at −105 mV, as a function of test voltage pulses. The relationships obtained from averaged data are fitted with a Boltzmann equation, and fit parameters are reported in Table 1. The activation curves for coexpressed p.T82A and p.G190S are intermediate between the relationships for p.G190S and p.T82A expressed alone (Color figure online)

Because p.T82A and p.R453W have apparently little effect on chloride currents, we wondered whether G190S may exert a dominant-like effect in the heterozygous carrier. We thus performed cotransfection experiments with the same amount (5 µg) of p.G190S and p.T82A plasmids in an attempt to recapitulate the heterozygous condition. Chloride currents were recorded using the high-chloride intracellular solution, which allow to clearly distinguish kinetics of the two mutants (Fig. 4). The chloride currents generated in cells transfected with 5 µg of p.G190S alone are also shown for comparison (Fig. 4a). Chloride currents, generated by p.G190S, slowly activated at positive voltages due to the shift of voltage dependence of activation. In addition, we observed slowly activating inward chloride currents at very negative voltages (<−100 mV). In cotransfected cells, chloride currents display properties reassuming both p.T82A and p.G190S chloride currents, including deactivating currents at negative voltages, inward chloride currents at negative voltages, and slowly activating outward currents at positive voltages (Fig. 4b). Importantly, the algebraic sum of p.T82A and p.G190S chloride current densities was quite superimposed to the chloride current density resulting from cotransfection (Fig. 4c, d). The voltage dependence of open probability of chloride currents recorded in cotransfected cells (p.T82A and p.G190S) was also in between the voltage dependences of each channel mutants expressed alone (p.T82A or p.G190S) (Fig. 4e). Very similar results were obtained with cotransfection of p.R453W and p.G190S (Online Resource 6).

### Quantitative Analysis of ion Channel Genes Expression in MC Patients Muscle Biopsies

**Fig. 5:**
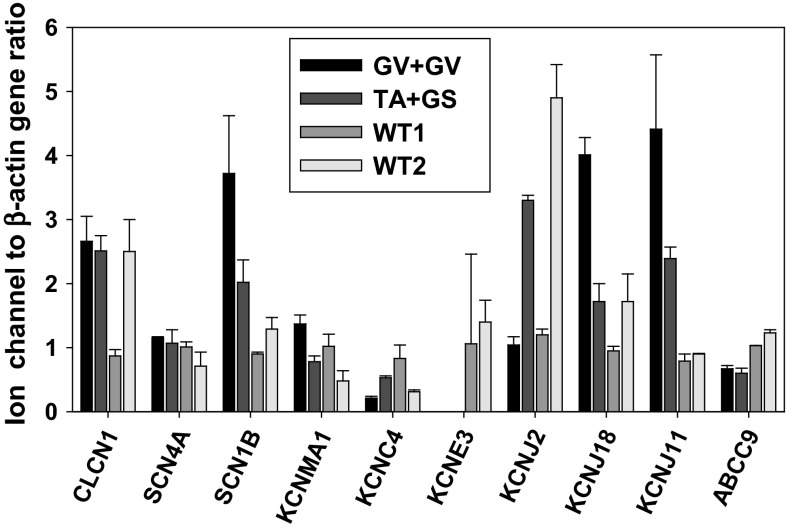
Quantitative gene expression of selected ion channels in biopsies of MC and control individuals. The transcript level is normalized with respect to *ACTB* housekeeping gene encoding β-actin. Each bar represents the mean ± SEM of triplicate measures in each bioptic sample. The *Vastus Lateralis* muscle biopsies are from the index myotonic patients of family 3 carrying p.G270V in homozygosis and of family 1 carrying p.T82A and p.G190S in compound heterozygosis. They are compared to biopsies of two control individuals

In order to better clarify the genotype–phenotype relationship, we performed a quantitative RTPCR analysis of a number of gene transcripts encoding sarcolemma ion channels in open *Vastus Lateralis* muscle biopsies from family 1 (carrying p.T82A and p.G190S) and family 3 (carrying p.G270V in homozygosis) probands, compared to two controls. The four individuals were male, aged 37–49 years. Exploratory ion channels and their properties are described in Tables (Online Resources 1 and 7). The exploratory genes do not constitute an exhaustive list of skeletal muscle ion channels, but all are involved in action potential propagation and/or have been linked to myotonia or periodic paralysis, as detailed in Online Resource 7. The results show that expression of ClC-1 mRNA was similar between MC and control individuals, excluding alterations in *CLCN1* expression as a contributor to myotonia in these patients (Fig. 5). Similarly, we observed no class effect between myotonic patients and controls for the voltage-dependent hNav1.4 sodium channel α-subunit (*SCN4A* gene), the large-conductance Ca^2+^-activated K^+^ channel (*KCNMA1* gene, Slo1 protein), the voltage-dependent K^+^ channel Kv3.4 (*KCNC4* gene), the sulfonylurea receptor type 2A (SUR2A protein, *ABCC9* gene), and two inward-rectifier K^+^ channels, Kir2.1 (*KCNJ2* gene) and Kir2.6 (*KCNJ18*). On the other hand, both MC biopsies showed a greater expression of the sodium channel β1 auxiliary subunit (*SCN1B* gene) and ATP-sensitive, inward-rectifier K^+^ (KATP) channel Kir6.2 (*KCNJ11* gene). The more striking observation was the total lack of MinK-related peptide 2 (MiRP2, *KCNE3* gene) expression in the MC biopsies.

## Discussion

Myotonia congenita (MC) is a musculoskeletal disorder whose identity firstly emerged with the seminal studies conducted by Bryant and colleagues, who described deficient muscle chloride conductance (Bryant 1969; Bryant and Morales-Aguilera 1971). The first *CLCN1* mutations for both autosomal recessive and dominant MC were discovered in the early 1990s (Koch et al. 1992; George et al. 1993). Although this specific form of myotonia is quite rare, researchers have learnt much more about the clinical, pathogenic, and molecular genetic aspects through various studies performed over the last 20 years (Imbrici et al. 2015). While it is well established that MC pathology is sustained by mutations in the *CLCN1* gene, a number of studies have highlighted a great clinical variability, even among patients carrying the same mutation. To date, more than 200 mutations have been detected in this gene, either in the dominant or in the recessive form of MC (Lossin and George 2008). It has been hypothesized that the clinical variability may be attributed to several factors as different expressivity, incomplete penetrance, impact of mutant alleles on wild-type channel proteins, allelic expression, or intrinsic variability of channel dysfunction (Duno et al. 2004).

Clinical myotonia can stem from many different causes. Natural history, inheritance trait, and disease aspects are relevant clues to distinguish different clinical entities. Age at onset, specific symptoms (i.e., predominant/transient weakness), and temperature effects may help in MC differential diagnosis, as well as neurophysiological or molecular results (Fournier et al. 2004; Lossin and George 2008; Heatwole et al. 2013). In this study, based on these criteria, MC was diagnosed in four patients. The molecular analysis confirmed the presence in these patients of four variants in the *CLCN1* gene, one of which has never been reported and two others have not been functionally characterized yet.

The p.G190S mutation in exon 5 is a known mutation, first described in a large consanguineous Arab family (Shalata et al. 2010), in which heterozygous individuals were asymptomatic or mildly affected, whereas homozygous individuals were severely ill. This mutation also appears widespread in Italy and has already been functionally characterized (Ulzi et al. 2012; Brugnoni et al. 2013; Desaphy et al. 2013). Its main effect consists in a dramatic shift of the open probability voltage dependence toward very positive voltages, resulting in nearly zero chloride current within the physiological voltage range of sarcolemma (Desaphy et al. 2013). It occurs indeed in a well-conserved motif in ClC-1 helix D, which is thought to play a critical role in the chloride ion pathway (Fahlke et al. 2001). In this cohort of MC patients, p.G190S was detected in three individuals from two unrelated families. The absence of neuromuscular disorders in their relatives suggests a recessive mode of inheritance; accordingly, these patients were compound heterozygous carrying also the p.T82A or p.R453W ClC-1 variants. Surprisingly, the latter variants displayed chloride currents very similar to WT channels in mammalian cell lines, in terms of amplitude, kinetics, and voltage dependence, thereby leaving their pathogenicity an open question.

The T82 residue is located at the N terminus in the intracellular side of the channel, far from the conducting pathway and the dimer interface. The residue is quite conserved among species, but shows variability among human CLC protein isoforms (see Online Resource 2). This variant was recently reported in two other Italian individuals and was predicted to be benign using the MutPred software (Brugnoni et al. 2013; Ulzi et al. 2014). Altogether, these observations argue for a likely weak role of T82 in ClC-1 function and, consequently, in the MC pathogenesis. The R453 residue is relatively more conserved among CLC proteins (see Online Resource 2). It is located in the extracellular loop between L and M segments on the extracellular side of the channel. The p.R453W was also previously reported and predicted as possibly disease causative (Brugnoni et al. 2013). Nevertheless, we found no effect of p.R453W on heterologously expressed chloride current properties. Importantly, p.G190S did not appear to exert any dominant-negative effect on p.T82A or p.R453W in coexpression studies, because the chloride currents generated in cells coexpressing p.G190S and the allelic mutant were similar to the computed sum of chloride currents measured in cells transfected with p.G190S alone or allelic mutant alone. Like p.T82A and p.R453W, other MC variants have been shown to produce chloride currents very similar to WT, including p.F167L and p.R105C (Desaphy et al. 2013). In addition, the quantification of *CLCN1* gene transcript in the muscle biopsy of family 1 proband suggests that changes in ClC-1 expression are likely not involved in the determination of myotonia, at least for p.G190S and p.T82A mutations. The mechanism by which such variants contribute to the clinical manifestation of myotonia remains unclear.

The p.G270V mutation was found homozygous in a patient with a positive family history. As previously mentioned, considering that two recessive mutations must be present in a single individual to induce myotonia and that myotonic symptoms were referred only in patient’s mother and maternal grandmother, we hypothesize that his father could have been an asymptomatic carrier of p.G270V, whereas his mother and maternal grandmother may harbor p.G270V associated with another *CLCN1* mutation. Being the proband relatives unavailable for molecular analysis, we are not able, up to date, to establish whether the p.G270V is a cause of a dominant or of a recessive form of MC in this family. However, the early onset and severity of symptoms could suggest a recessive MC. To our knowledge, this is the first report of the p.G270V mutation. The mutation is located in exon 7 and occurs in a well-conserved motif of the transmembrane G helix, close to the conducting pathway (see Online Resource 2). Other neighboring mutations have been associated with myotonia, including p.C271R, p.V273M, p.G276D, p.C277R, and p.C277Y (Fialho et al. 2007; Weinberger et al. 2012; Brugnoni et al. 2013). The last two have been shown to profoundly disrupt ClC-1 channel function (Weinberger et al. 2012). Using MutPred software (http://mutpred.mutdb.org/), p.G270V was scored with a 0.78 probability to be deleterious. Accordingly, p.G270V drastically shifts the channel voltage dependence in tsA201 cells, which likely accounts for a dramatically reduced chloride conductance and consequent myotonia in the homozygous family 3 proband.

One of the recurrent themes regarding MC is the variable clinical presentation. Among the various hypotheses to explain such a variability, one possibility encompasses the expression of disease modifiers in myotonic patients. Using RT-PCR, we analyzed the expression of selected ion channel subunits involved in action potential propagation and/or previously linked to muscle excitability disorders. Although quantification of ion channel transcripts was performed in only two MC patients, the results suggest that myotonia might be associated with changes in expression of voltage-dependent Na^+^ and K^+^ channels, and ATP-sensitive K^+^ channels. The β1 subunit (*SCN1B* gene) may affect the membrane surface expression and voltage dependence of the Na^+^ channel α-subunit and possibly of the repolarizing K^+^ voltage-dependent channels, thereby modulating cell excitability (Desaphy et al. 2001; Brackenbury and Isom 2011; Marionneau et al. 2012). Strikingly, the auxiliary subunit of voltage-gated K^+^ channels, MiRP2, is totally lacking in muscle biopsies of myotonic patients. Coassembly with MiRP2 modifies the voltage dependence of Kv3.4, converting the channel to a subthreshold-activating channel that contributes to skeletal muscle resting potential (Abbott et al. 2001). The KATP channels link metabolism to muscle activity and may exert a significant myoprotective action under prolonged muscle activity, which may occur during myotonia due to delayed relaxation (Tricarico et al. 2006; Flagg et al. 2010). Although the limited number of analyzed biopsies impedes a generalization of the results, we hypothesize that extending such analysis to a larger number of samples and of exploratory genes may provide relevant information to improve our understanding of the myotonia etiopathogenesis and may help in the identification of appealing druggable targets.

In conclusion, the three new *CLCN1* variants can be added to the growing database of MC-associated mutations. Functional studies support the pathogenicity of p.G270V, whereas the mechanisms by which p.T82A and p.R453W may cause the disease remained elusive. Other studies are necessary to definitely classify these two variants as pathogenic mutations (Tang and Chen 2011). In addition, a possible identification of disease modifiers in MC muscle biopsies could help to elucidate the disease mechanisms and broaden therapeutic options. The therapy for patients suffering from MC is at the moment purely symptomatic, consisting in the use of sodium channel blockers such as mexiletine. It is expected that the understanding of the various disease mechanisms linked to *CLCN1* mutations could help the development of targeted drugs with improved efficacy and tolerability.

## Electronic supplementary material

Supplementary material 1 (PDF 1096 kb)
