# Supplementary material for: Clinical, Molecular, and Functional Characterization of CLCN1 Mutations in Three Families with Recessive Myotonia Congenita
Source: Neuromolecular Med. 2015 May 26;17(3):285–96. doi: 10.1007/s12017-015-8356-8 (PMC4534513; doi:10.1007/s12017-015-8356-8)
Supplement: Supplementary file 1 — Supplementary material 1 (PDF 1096 kb) [file 12017_2015_8356_MOESM1_ESM.pdf]

Online Resource 1. NeuroMol Med

Portaro S, Altamura C, Licata N, Camerino GM, Imbrici P, Musumeci O, Rodolico C, Conte Camerino D, Toscano A, & Desaphy J-F. Clinical, molecular and functional characterization of CLCN1 mutations in three families with recessive Myotonia Congenita.

University of Bari Aldo Moro, Bari, Italy. E-mail: jeanfrancois.desaphy@uniba.it

University of Messina, Messina, Italy. E-mail: atoscano@unime.it

List of genes quantified by real-time PCR with relative Life Technologies assay IDs.

Gene name	Protein name	Assay IDs
CLCN1	ClC-1 voltage-sensitive chloride channel	Hs00892505_m1
SCN4A	Nav1.4 voltage-gated sodium channel α subunit	Hs01109480_m1
SCN1B	Voltage-gated sodium channel auxiliary β1 subunit	Hs03987893_m1
KCNMA1	Large conductance Ca^{2+}-activated K^{+} channel α subunit	Hs01119498_m1
KCNC4	Kv3.4 voltage-gated K+ channel α-subunit	Hs00428198_m1
KCNE3	MinK-Related Peptide 2 (MiRP2)	Hs00538801_m1
KCNJ2	Kir2.1 inwardly-rectifying K+ channel	Hs00265315_m1
KCNJ18	Kir2.6 inwardly-rectifying K+ channel	Hs00253248_s1
KCNJ11	Kir6.2 inwardly-rectifying K+ channel	Hs00265026_s1
ABCC9	SUR2A ATP-binding cassette	Hs01072316_m1
HPRT1	hypoxanthine phosphoribosyltransferase 1	Hs02800695_m1
ACTB	β-actin	Hs99999903_m1
B2M	β2-microglobulin	Hs00984230_m1

Online Resource 2. NeuroMol Med

Portaro S, Altamura C, Licata N, Camerino GM, Imbrici P, Musumeci O, Rodolico C, Conte Camerino D, Toscano A, & Desaphy J-F. Clinical, molecular and functional characterization of CLCN1 mutations in three families with recessive Myotonia Congenita.

University of Bari Aldo Moro, Bari, Italy. E-mail: jeanfrancois.desaphy@uniba.it

University of Messina, Messina, Italy. E-mail: atoscano@unime.it

Amino acid alignment of CLC proteins with Clustal 2.2

```
Species-specific ClC-1:
Human  (T82)     IYGHHKEQFSDREQDIGMPKKTG----------------SSSTVDSKDEDHYSKCQDCIH 104
Rat    (T82)     IYGHHKEQYSYQAQDRGIPKKTD----------------SSSTVDSLDEDHYSKCQDCVH 104
Mouse  (M82)     IYGHQKEQYSYKAQDGGMPKKMG----------------SSSTMDSLDEDHYSKCQDCVH 104
Dog    (M12)     ----------DKEQDTGMSKKMG----------------SSESMDSKDEDHYSKCQGCVR 34
Human CLC members:
ClC-2  (R56)     IR------LGGPEPWKGPPSSRA----------------APELLEYG-RSRCARCRVCSV 77
ClC-Ka (F11)     -----------MEELVGLREGFS----------------GDPVTLQELWGPCPHIRRAIQ 33
ClC-Kb (S11)     -----------MEEFVGLREGSS----------------GNPVTLQELWGPCPRIRRGIR 33
ClC-3  (V82)     NGGSINSSTHLLDLLDEPIPGVG--------TYDDFHTIDWVREKCKDRERHRRINSKKK 112
ClC-4  (V24)     NAGAMSGSGNLMDFLDEPFPDVG--------TYEDFHTIDWLREKSRDTDRHRKITSKSK 54
ClC-5  (V11)     -----------MDFLEEPIPGVG--------TYDDFNTIDWVREKSRDRDRHREITNKSK 41
ClC-6  (X)       ---------ETQEEEDEILP-------------------RKDYESLDYDRCINDPYLEV 64
ClC-7  (D72)     SALFRVGHMSSVELDDELLDPDMDPPHPFPKEIPHNEKLLSLKYESLDYDNSENQLFLEE 110

Species-specific ClC-1:
Human  (G270)    ICAAVLSKFMSVFCG-------VYEQPYYYSDILTVGCAVGVGCCFGTPLGGVLFSIEVT 293
Rat    (G270)    ICAAVLSKFMSMFSG-------VYEQPYYYTDILTVGCAVGVGCCFGTPLGGVLFSIEVT 293
Mouse  (G270)    ICAAVLSKFMSMFSG-------VYEQPYYYTDILTVGCAVGVGCCFGTPLGGVLFSIEVT 293
Dog    (G200)    ICAAVLSKFMSMFCG-------VYEQPYYYTDMLTVGCAVGVGCCFGTPLGGVLFSIEVT 223
Human CLC members:
ClC-2  (A243)    MCAALLSKFLSLFGG-------IYENESRNTEMLAAACAVGVGCCFAAPIGGVLFSIEVT 266
ClC-Ka (A204)    MIAAYLGRVRTTTIG-------EPENKSKQNEMLVAAAAVGVATVFAAPFSGVLFSIEVM 227
ClC-Kb (A204)    MMAAYLGRVRTTTIG-------EPENKSKQNEMLVAAAAVGVATVFAAPFSGVLFSIEVM 227
ClC-3  (A317)    CCGNIFSYLFPK----------YSTNEAKKREVLSAASAAGVSVAFGAPIGGVLFSLEEV 340
ClC-4  (A259)    CCGNFFSSLFSK----------YSKNEGKRREVLSAAAAAGVSVAFGAPIGGVLFSLEEV 282
ClC-5  (A246)    CCGNILCHCFNK----------YRKNEAKRREVLSAAAAAGVSVAFGAPIGGVLFSLEEV 269
ClC-6  (G245)    VVGAGLPQFQSISLRKIQFNFPYFRSDRDKRDFVSAGAAAGVAAAFGAPIGGTLFSLEEG 268
ClC-7  (G292)    VIAAGISQGRSTSLKRDFKIFEYFRRDTEKRDFVSAGAAAGVSAAFGAPVGGVLFSLEEG 315


Species-specific ClC-1:
Human  (R453)    LGQSAVWIHPRVNVVIIIFLFFVMKFWMSIVATTMPIPCGGFMPVFVLGAAFGRLVGEIM 502
Rat    (Q453)    LGQSAVWIHPQVNVVIIIILLFFVMKFWMSIVATTMPIPCGGFMPVFVLGAAFGRLVGEIM 502
Mouse  (Q453)    LGQSAVWLHPQVNVIIIIILLFFVMKFWMSIVATTMPIPCGGFMPVFVLGAAFGRLVGEIM 502
Dog    (R383)    LGRSAVWIHPRVNVIIIIFLFFIMKFWMSIVATTMPIPCGGFMPVFVLGAAFGRLVGEIM 432
Human CLC members:
ClC-2  (R428)    PSTSQAWNPPRANVFLTLVIFILMKFWMSALATTIPVPCGAFMPVFVIGAAFGRLVGESM 477
ClC-Ka (R395)    QHLWWEWYHPRFTIFGTLAFFLVMKFWMLILATTIPMPAGYFMPIFILGAAIGRLLGEAL 444
ClC-Kb (R395)    QHLWWEWYHPRFTIFGTLAFFLVMKFWMLILATTIPMPAGYFMPIFVYGAAIGRLFGETL 444
ClC-3  (X)       PAGIGVYSA-----IWQLCLALIFKIIMTVFTFGIKVPSGLFIPSMAIGAIAGRIVGIAV 545
ClC-4  (X)       PAGVGVYTA-----MWQLALALIFKIVVTIFTFGMKIPSGLFIPSMAVGAIAGRMVGIGV 487
ClC-5  (X)       PAGVGVYSA-----MWQLALTLILKIVITIFTFGMKIPSGLFIPSMAVGAIAGRLLGVGM 473
ClC-6  (G458)    AILQLFHQD-GTFSPVTLALFFVLYFLLACWTYGISVPSGLFVPSLLCGAAFGRLVAN-- 505
ClC-7  (G483)    SVVSLFHDPPGSYNPLTLGLFTLVYFFLACWTYGLTVSAGVFIPSLLIGAAWGRLFG--- 529
```

Online Resource 3. NeuroMol Med
Portaro S, Altamura C, Licata N, Camerino GM, Imbrici P, Musumeci O, Rodolico C, Conte Camerino D, Toscano A, & Desaphy J-F. Clinical, molecular and functional characterization of CLCN1 mutations in three families with recessive Myotonia Congenita.
University of Bari Aldo Moro, Bari, Italy. E-mail: jeanfrancois.desaphy@uniba.it
University of Messina, Messina, Italy. E-mail: atoscano@unime.it

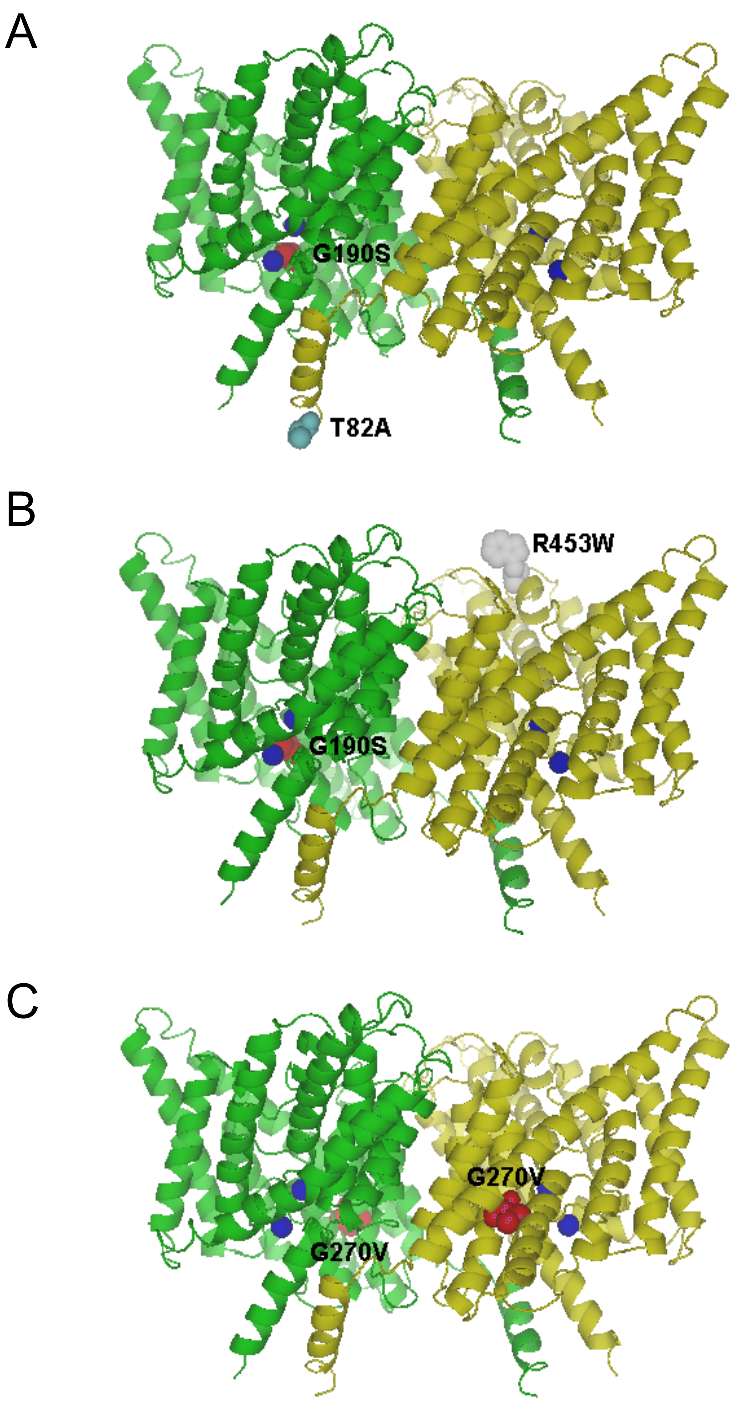

Three dimensional ribbon representation of the ClC-1 channel homodimer model based upon the structure of ClC-ec1 viewed front the plane of the membrane. The two subunits are shown in green and yellow with Cl- ions (blue spheres) located in the pore of each subunit. Myotonic mutations are shown in heterozygosity for p.G190S (red) and p.T82A (cyan) (A), p.G190S and p.R453W (grey) (B), and homozygosity for p.G270V (red in C).

Online Resource 3. NeuroMol Med

Portaro S, Altamura C, Licata N, Camerino GM, Imbrici P, Musumeci O, Rodolico C, Conte Camerino D, Toscano A, & Desaphy J-F. Clinical, molecular and functional characterization of CLCN1 mutations in three families with recessive Myotonia Congenita.

University of Bari Aldo Moro, Bari, Italy. E-mail: jeanfrancois.desaphy@uniba.it

University of Messina, Messina, Italy. E-mail: atoscano@unime.it

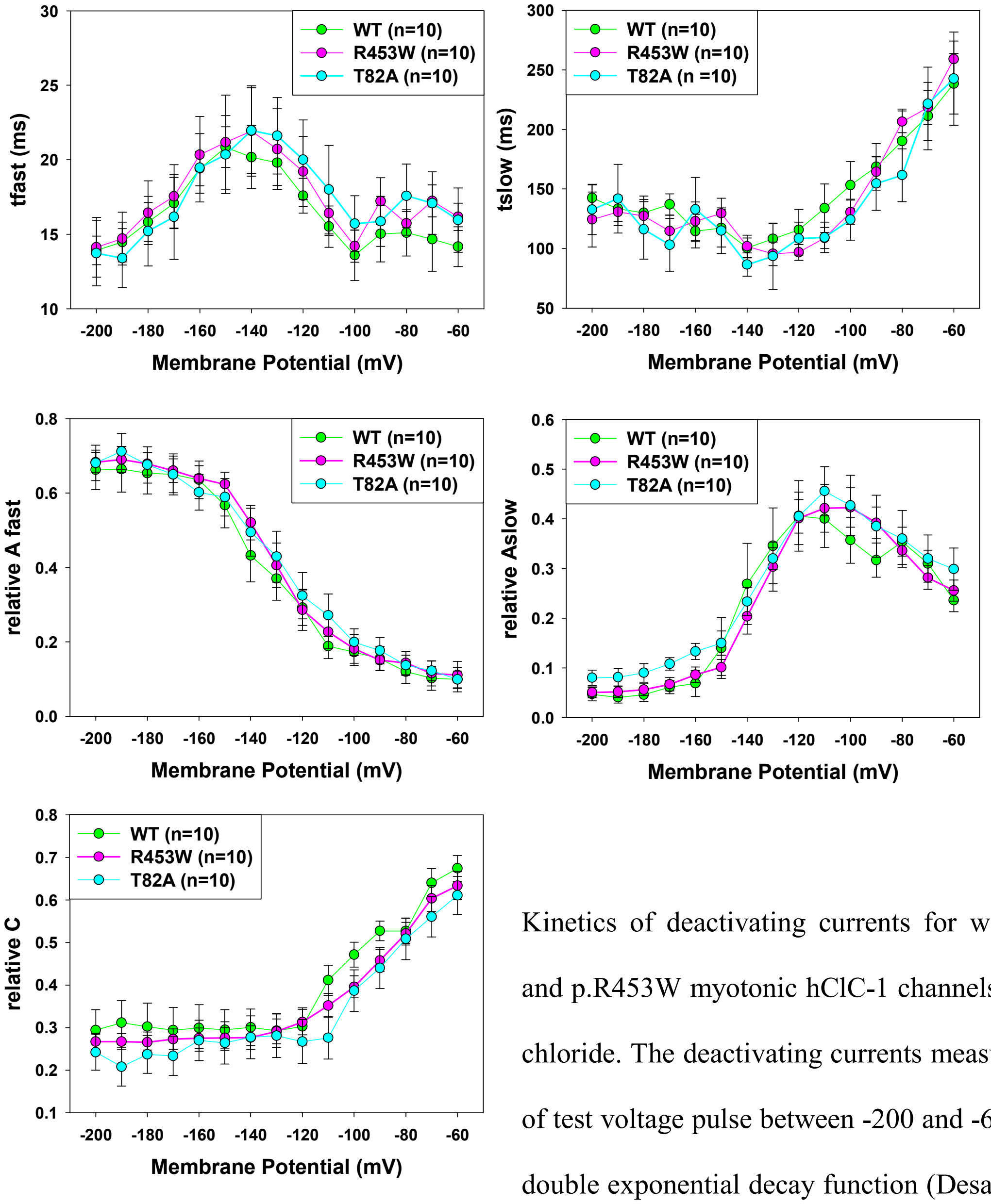

Kinetics of deactivating currents for wild-type and p.T82A and p.R453W myotonic hClC-1 channels in high intracellular chloride. The deactivating currents measured at the beginning of test voltage pulse between -200 and -60 mV were fit with a double exponential decay function (Desaphy et al. 2013). The fit parameters include a fast time constant (τ_{fast}, 10-ms range), a slow time constant (τ_{slow}, 100-ms range), and their respective contribution to total current (A_{fast}, A_{slow}), plus the contribution of residual steady state current (relative C).

Online Resource 5. NeuroMol Med

Portaro S, Altamura C, Licata N, Camerino GM, Imbrici P, Musumeci O, Rodolico C, Conte Camerino D, Toscano A, & Desaphy J-F. Clinical, molecular and functional characterization of CLCN1 mutations in three families with recessive Myotonia Congenita.
University of Bari Aldo Moro, Bari, Italy. E-mail: jeanfrancois.desaphy@uniba.it
University of Messina, Messina, Italy. E-mail: atoscano@unime.it

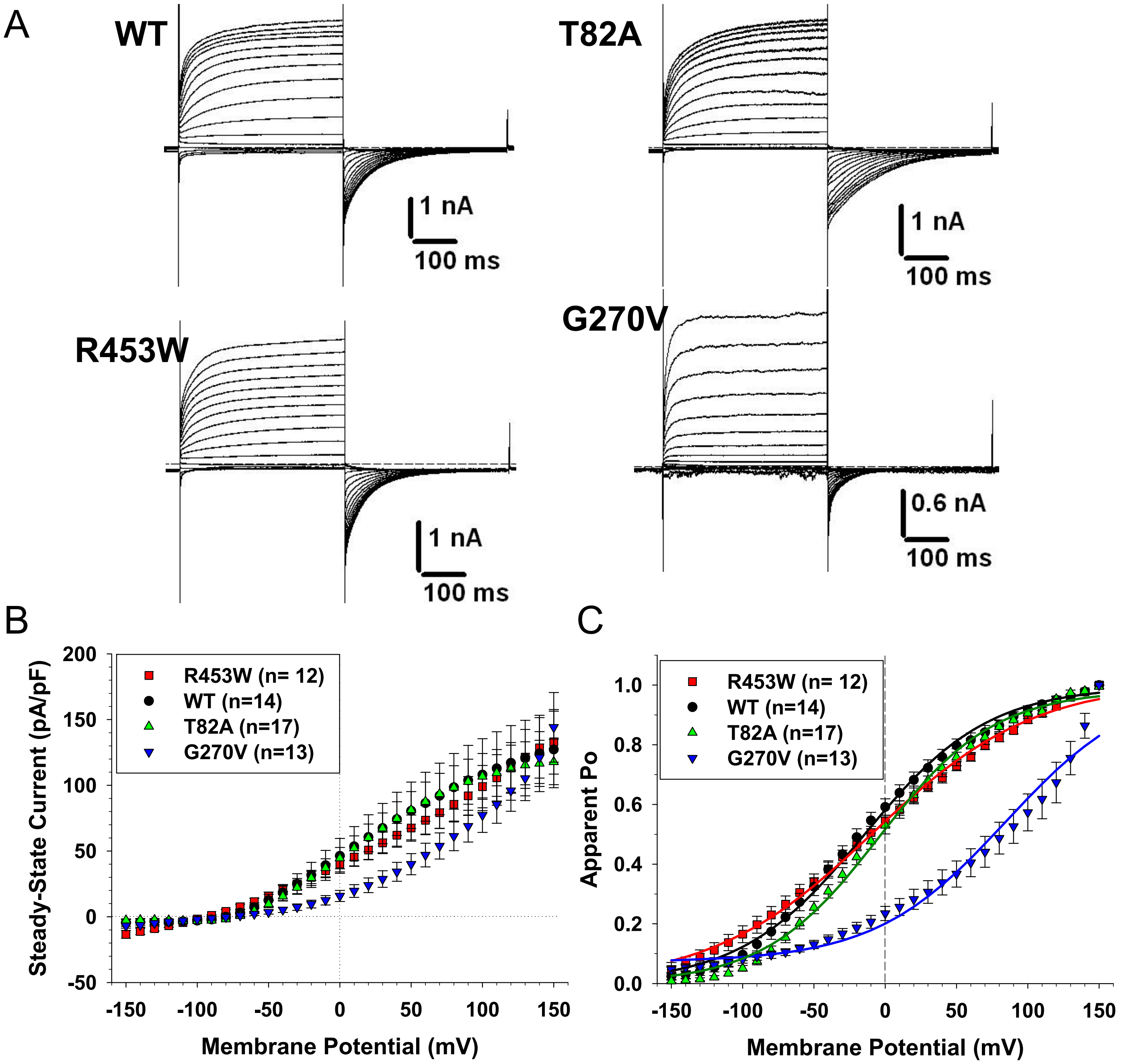

Chloride currents generated by wild-type hClC-1 channels and myotonic mutants in low intracellular chloride condition. **A)** Typical chloride currents recorded in HEK293 cells transfected with wild-type, p.T82A, p.R453W, and p.G270V hClC-1 variants. Cells were held at -95 mV and 400 ms voltage pulses were applied from -150 to +150 mV in 10-mV intervals every 3 seconds. For clarity only current traces obtained every 20 mV are shown. Chloride currents displayed similar kinetics, but p.G270V currents had reduced amplitude. **B)** The steady-state current density-voltage relationships show a reduced current density of p.G270V channels. **C)** The voltage dependence of activation were fit with a Boltzmann function (eq. 1). Fit parameters are reported in Table 1. Only p.G270V channels displayed a positively-shifted voltage dependence with respect to WT.

Online Resource 4. NeuroMol Med

Portaro S, Altamura C, Licata N, Camerino GM, Imbrici P, Musumeci O, Rodolico C, Conte Camerino D, Toscano A, & Desaphy J-F. Clinical, molecular and functional characterization of CLCN1 mutations in three families with recessive Myotonia Congenita.

University of Bari Aldo Moro, Bari, Italy. E-mail: jeanfrancois.desaphy@uniba.it

University of Messina, Messina, Italy. E-mail: atoscano@unime.it

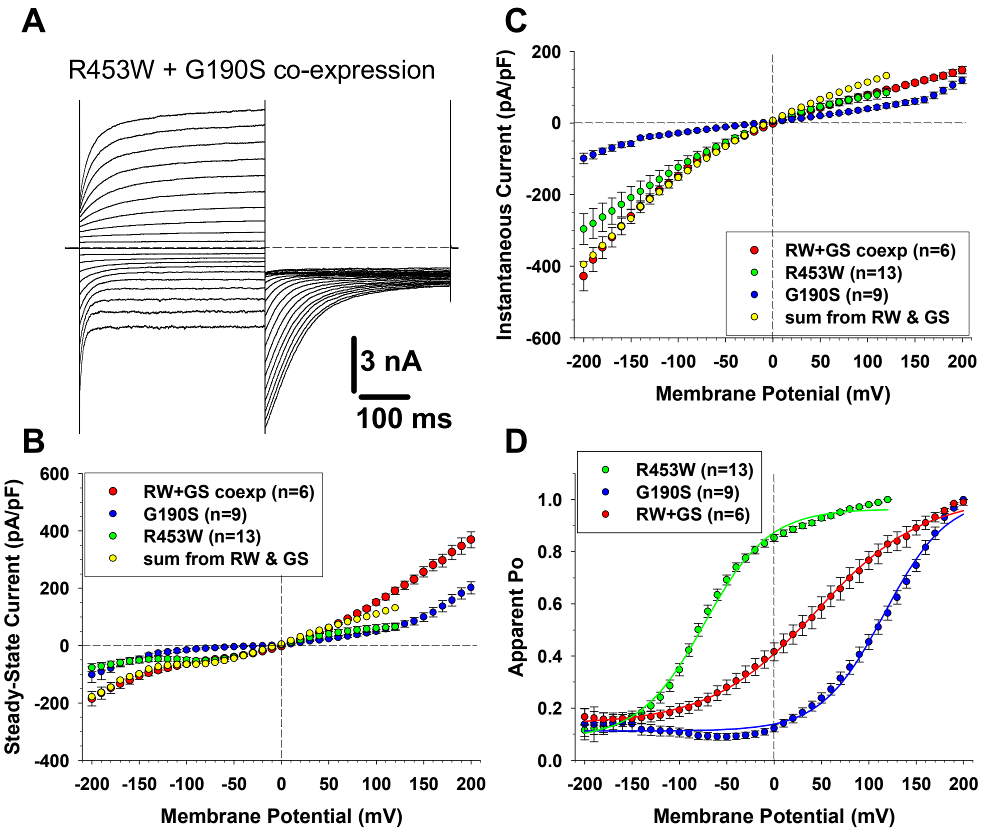

Chloride currents were recorded in tsA201 cells transfected with either p.G190S or p.R453W alone, or with both mutants together, using high intracellular chloride condition. **(A)** Family of chloride currents generated by cotransfected p.G190S and p.R453W mutants. The cell was held at 0 mV and voltage pulses were applied from -200 to +200 mV in 10-mV intervals every 3 seconds. For clarity only current traces obtained every 20 mV are shown. The mean I-V curves for instantaneous **(B)** and steady-state **(C)** currents are shown for p.R453W, p.G190S, and co-expressed p.G190S/p.R453W. In yellow are reported the algebraic sum of current densities calculated for p.G190S and p.R453W expressed alone. The yellow points are quite superimposed to the I-V relationship for co-expressed p.G190S + p.R453W. **(D)** The activation curves for co-expressed p.R453W + p.G190S is in between the relationships for p.G190S and p.R453W expressed alone. The Boltzmann fit parameters are reported in Table 1 of the manuscript.

Online Resource 5. NeuroMol Med

Portaro S, Altamura C, Licata N, Camerino GM, Imbrici P, Musumeci O, Rodolico C, Conte Camerino D, Toscano A, & Desaphy J-F. Clinical, molecular and functional characterization of CLCN1 mutations in three families with recessive Myotonia Congenita.

University of Bari Aldo Moro, Bari, Italy. E-mail: jeanfrancois.desaphy@uniba.it

University of Messina, Messina, Italy. E-mail: atoscano@unime.it

Description of ion channels genes quantified by real-time PCR.

Channel (gene)	Channel (dys)function	Some References
ClC-1 (*CLCN1*)	Voltage-sensitive chloride channel. Mutations cause Myotonia Congenita	Desaphy et al., *Exp Neurol* 248: 530-540, 2013
Nav1.4 (*SCN4A*)	Skeletal muscle sodium channel α-subunit. Mutations cause Paramyotonia Congenita, Sodium Channel Myotonias, hyperkalemic Periodic Paralysis, hypokalemic Periodic Paralysis type 2.<br>Is the molecular target of the antimyotonic drug mexiletine.	Desaphy et al., *Neurology* 57: 1849-1857, 2001
β1 (*SCN1B*)	Nav1.4 auxiliary subunit. Mutations cause genetic epilepsy with febrile seizures plus (GEFS)+	Wallace et al., *Nat Genet* 19: 366-370, 1998; Brackenbury and Isom, *Front Pharmacol* 2: 53, 2011
Slo1 (*KCNMA1*)	Large conductance Ca^{2+}-activated K+ channel. Is one of the target for the antimyotonic drug acetazolamide	Tricarico et al., *FASEB J* 18: 760-761, 2004.
Kv3.4 (*KCNC4*)	Voltage-gated K+ channel α-subunit. Contributes to action potential repolarization and resting potential	Abbott et al., *Cell* 104: 217–231, 2001
MiRP2 (*KCNE3*)	voltage-gated K+ channel auxiliary subunit. Contributes to resting potential	Abbott et al., *Cell* 104: 217–231, 2001
Kir2.1 (*KCNJ2*)	Inwardly-rectifying K+ channel. Mutations cause Andersen-Tawil syndrome including periodic paralysis	Tristani-Firouzi and Etheridge, *Pflugers Arch* 460: 289-294, 2010.
Kir2.6 (*KCNJ18*)	Inwardly-rectifying K+ channel. Mutations may be linked to thyrotoxic and sporadic periodic paralysis, although this has been recently challenged	Cheng et al., *J Biol Chem* 286: 27425-27435, 2011; Kuhn et al., J Neurol Neurosurg Psychiatry 2015
Kir6.2 (*KCNJ11*)	ATP–sensitive inwardly-rectifying K+ channel.<br>Channel activity is modified in hypokalemic Periodic Paralysis	Tricarico et al., *J Clin Invest* 103: 675-682, 2001; Flagg et al., *Physiol Rev* 90: 799-829, 2010
